# The transcription factor FOXQ1 in cancer

**DOI:** 10.1007/s10555-025-10240-y

**Published:** 2025-01-08

**Authors:** Stefan Koch

**Affiliations:** 1 Wallenberg Centre for Molecular Medicine (WCMM), Linköping University, Linköping, Sweden; 2 Department of Biomedical and Clinical Sciences (BKV), Linköping University, BKV/MMV – Plan 13, Lab 1, 581 85 Linköping, Sweden

**Keywords:** Forkhead box, Transcription factor, Metastasis, Gene regulation, Epithelial-to-mesenchymal transition

## Abstract

FOXQ1 is a member of the large forkhead box (FOX) family of transcription factors that is involved in all aspects of mammalian development, physiology, and pathobiology. FOXQ1 has emerged as a major regulator of epithelial-to-mesenchymal transition and tumour metastasis in cancers, especially carcinomas of the digestive tract. Accordingly, FOXQ1 induction is recognised as an independent prognostic factor for worse overall survival in several types of cancer, including gastric and colorectal cancer. In this review article, I summarise new evidence on the role of FOXQ1 in cancer, with a focus on molecular mechanisms that control FOXQ1 levels and the regulation of FOXQ1 target genes. Unravelling the functions of FOXQ1 has the potential to facilitate the development of targeted treatments for metastatic cancers.

## Forkhead box transcription factors

Forkhead box (FOX) proteins constitute one of the largest mammalian transcription factor families, encompassing 50 FOX proteins in humans and 44 family members in mice [1, 2]. These are grouped into 19 subfamilies (A through S) based on sequence similarity of their eponymous, DNA-binding forkhead box domain. Whereas some FOX transcription factors, such as members of the O, J, and P subfamilies, are broadly expressed across different tissues, others exhibit a much more restricted expression pattern and may thus exert tissue-specific functions [1]. FOX proteins play essential roles in embryogenesis and normal tissue homeostasis, and their dysfunction is associated with numerous disorders, including developmental defects and cancers [3, 4]. Some FOX family members have been studied in exceptional detail. For example, FOXM1 is a major oncogene in various cancers such as colorectal, breast, and lung cancer, and is known to contribute to tumour metastasis and treatment resistance [5]. FOXA1-3, as another example, are prominent pioneer factors that locally open chromatin to allow gene transcription by other transcription factors, and their dysfunction is similarly associated with severe diseases, particularly prostate and breast cancer [6]. However, most other FOX family members have received considerably less attention despite their often critical roles in health and disease. In this article, I review the functions of the transcription factor FOXQ1, which has emerged as another FOX protein with major functions in cancers.

## The structure of FOXQ1 and its function in normal physiology

**Fig. 1:**
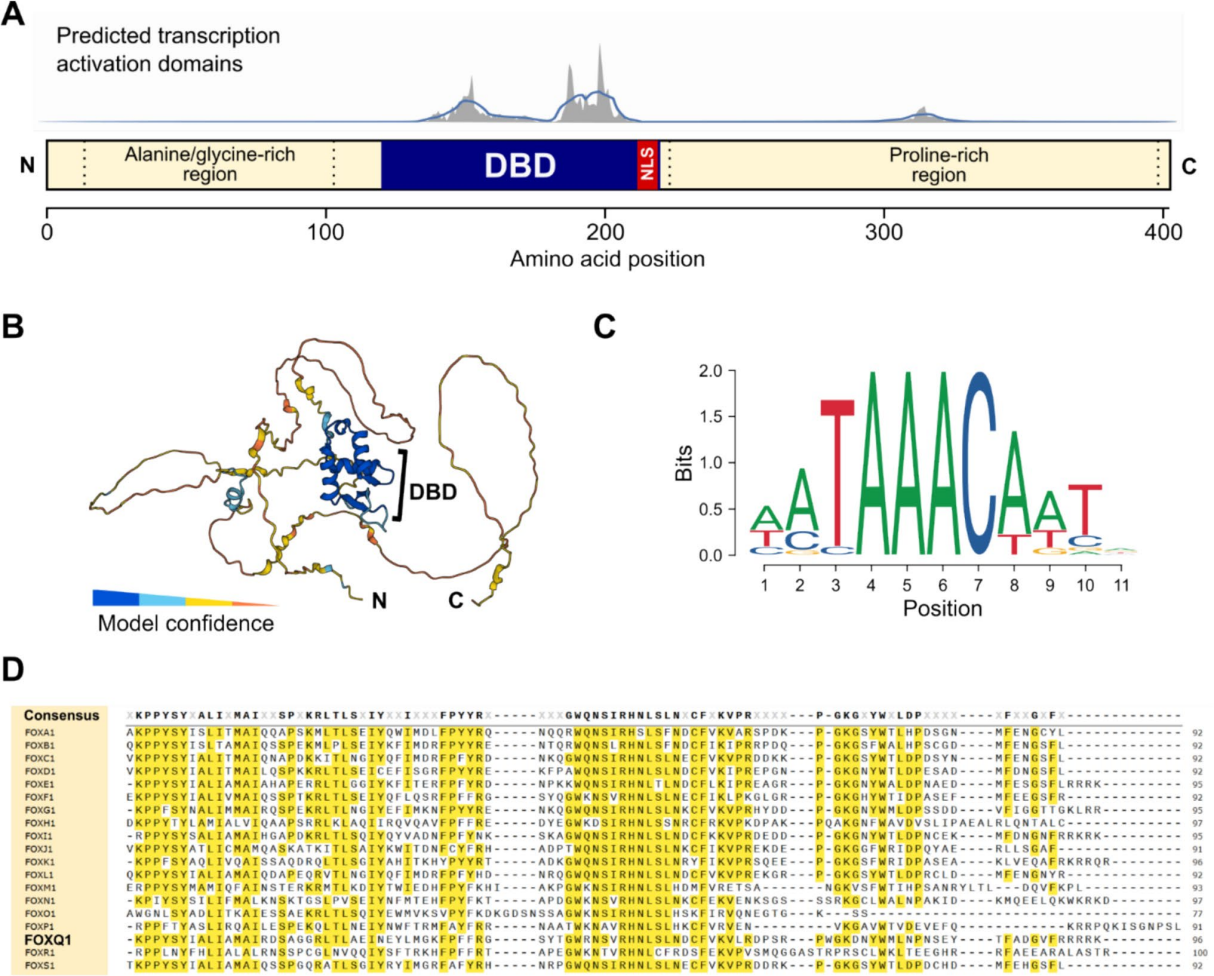
The structure of FOXQ1. **A** Domain organisation of the human FOXQ1 protein. The prediction of possible acidic transcription activation domains was done in ADpred [10]. DBD: DNA-binding domain; NLS: nuclear localisation sequence. **B** Predicted structure of human FOXQ1 (AlphaFold model AF-Q9C009-F1). The highly conserved DBD is flanked by disordered N and C-termini. **C** DNA recognition motif of rat Foxq1 (JASPAR ID MA0040.1), whose DBD is identical to the human protein. **D** Sequence alignment of the DBD of representative members of all 19 FOX subfamilies. The consensus sequence is shown on top, and conserved residues are highlighted in yellow. The alignment was performed in Clustal Omega [130]

The FOX family member FOXQ1, then known as Hepatocyte Nuclear Factor 3 / Forkhead Homolog 1 (HFH1), was first identified in 1993 as a tissue-restricted transcription factor [7]. The single-exon, 2.6kb human *FOXQ1* gene is located on chromosome 6 and encodes a 403 amino acid protein. As with the other FOX family members, the FOXQ1 protein consists of the central forkhead box domain flanked by long, largely disordered N and C-termini that mediate its specific functions (Fig. 1A, B). The forkhead box domain of FOXQ1 is highly conserved across species and binds to the canonical forkhead recognition motif RYAAAYA [8] (Fig. 1C, D). Moreover, Mitchell et al. proposed that the forkhead box of FOXQ1 acts as a transactivation domain by recruiting transcription co-factors to target gene promoters [9], which is supported by *in silico* prediction of transcription activation domains in the FOXQ1 protein [10] (Fig. 1A). In contrast, the contribution of the N and C-termini to gene regulation remains elusive as they contain no apparent functional domains, although it has been noted that these regions are enriched for alanine/glycine and proline residues, respectively [11], and may thus have discrete functions. Consistently, we observed that the N and C-termini of FOXQ1 differentially contribute the regulation of transcription activation and repression, with the C-terminus being mainly involved in gene repression [12].

In normal mammalian physiology, FOXQ1 appears to be mostly involved in embryogenesis. Studies in mice revealed that *Foxq1* is expressed throughout embryonic development, and that its loss-of-function causes increased embryonic lethality in some genetic backgrounds [13, 14]. On the other hand, *Foxq1* expression levels are very low in tissues of adult animals except for the stomach and bladder. Accordingly, the major pathological phenotype of adult *Foxq1*-deficient mice is impaired mucus and acid secretion in the stomach, which is thought to be caused by incomplete cell differentiation in the gastric mucosa [14, 15]. Defective cell differentiation also explains the most notable feature of *Foxq1* mutant mice, namely their shiny, satin fur [13, 16, 17]. It was found that *Foxq1* is expressed in the medulla of the hair follicle [17], and that *Foxq1* mutations result in defective patterning of the hair shaft, causing the fur to be more refractive [16]. Additional roles that have been ascribed to FOXQ1 include the regulation of glucose and lactate metabolism in various cells types [18–20], as well as the control of cell death in alveolar epithelial cells [21], although these functions are less well understood. Altogether, these findings suggest that FOXQ1 exerts minor specific functions in diverse tissues and contexts, but that its loss-of-function is well tolerated in the adult organism.

## Expression of FOXQ1 in different cancers and its association with disease progression

**Fig. 2:**
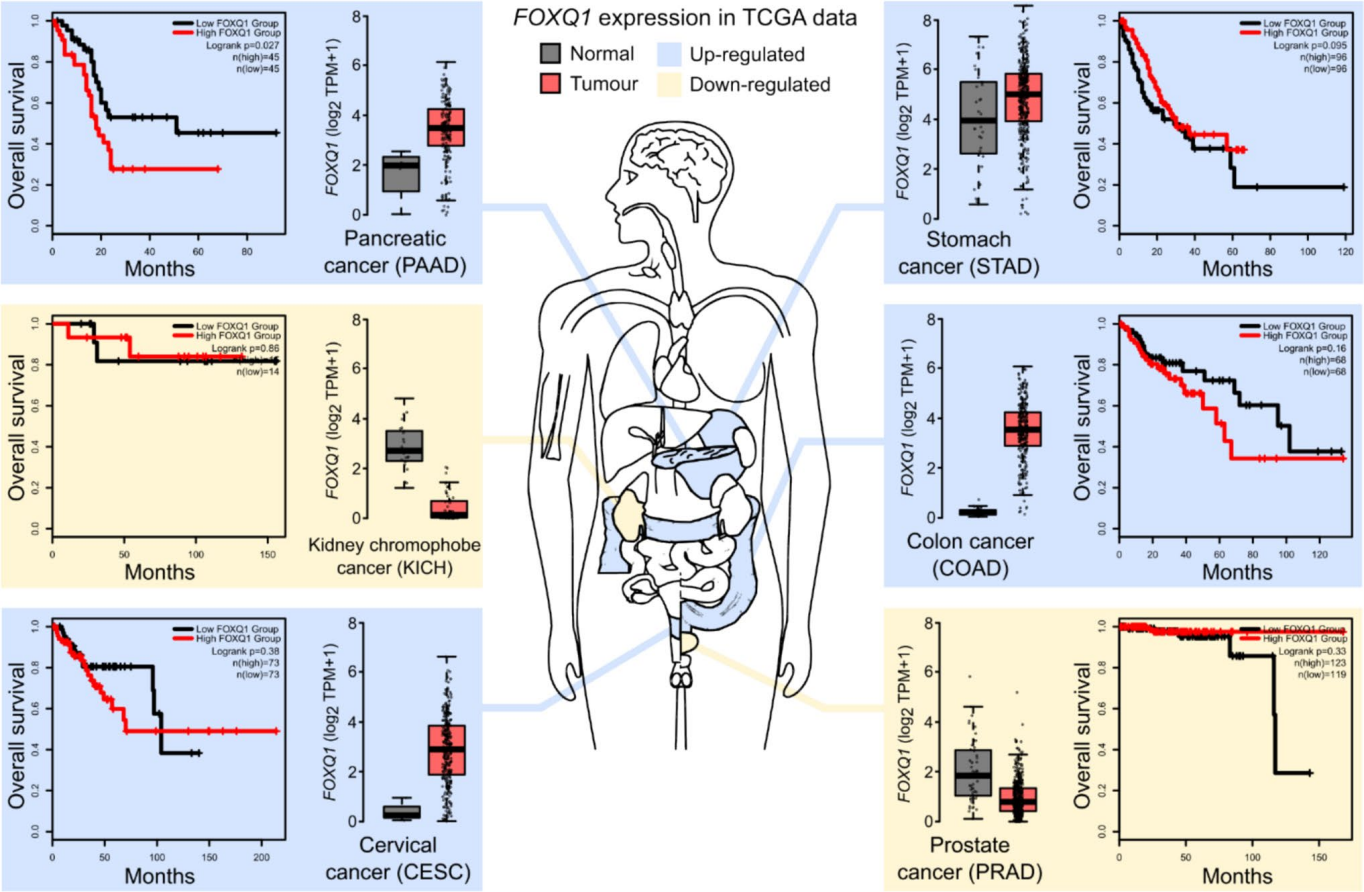
Altered expression of *FOXQ1* in cancer. The bar graphs depict FOXQ1 mRNA levels in selected cancers from The Cancer Genome Atlas (TCGA), compared to normal tissue samples. The TCGA dataset designation is shown in parentheses. TPM: Transcripts per million. The corresponding survival data of patients with cancer were stratified by quartiles, and analysed using the Logrank test. Data were retrieved from and plotted in GEPIA 2 [131]

While the contribution of FOXQ1 to normal physiology and tissue homeostasis appears to be limited, this transcription factor has gained considerable attention in the context of cancer biology. In 2010, Kaneda and colleagues reported that *FOXQ1* expression is strongly increased in tissue samples from patients with colorectal cancer (CRC), and that its induction inhibited the apoptosis of CRC cell lines in the presence of chemotherapeutic drugs [22]. Subsequent studies confirmed and expanded on these findings, showing that altered expression of *FOXQ1* can be observed in numerous types of cancer (Fig. 2). Cancers with increased *FOXQ1* levels include, especially, carcinomas of the colon [22–25], but also epithelia-derived cancers of the lung [26], the stomach [27], the pancreas [28], and the oesophagus [29]. In general, high *FOXQ1* expression appears to be associated with a poor clinical outcome, and has been identified as an independent prognostic marker for worse survival in colorectal, pancreatic, lung, gastric, and hepatocellular carcinomas (HCC) [26–28, 30, 31]. There are notable exceptions from this rule. Several studies reported increased *FOXQ1* expression in breast cancer cells [32–34]. However, in some subtypes of breast cancer, namely HER2-positive and luminal breast cancer, *FOXQ1* expression levels are lower compared to healthy tissue and triple-negative breast cancer (TNBC) [35]. In these cancers, high *FOXQ1* expression was found to be associated with more favourable clinical outcomes. Lower expression of *FOXQ1* was also observed in remote metastases of cutaneous melanomas, and FOXQ1 over-expression reduced melanoma tumour growth in mouse xenograft models [36]. Public cancer data from sources such as The Cancer Genome Atlas (TCGA) suggest that low FOXQ1 expression levels may similarly be associated with worse survival in other types of cancer, including prostate and urothelial cancer (see, e.g., the Human Protein Atlas pathology resource [37]); however, these associations have not been explored to date. Taken together, these findings outline a dual role of FOXQ1 in cancer: in most types of cancer, especially carcinomas, FOXQ1 is induced and promotes disease progression. However, FOXQ1 can apparently also function as a tumour suppressor in some types of cancer, and its loss in these cancers contributes to a worse prognosis. Possible molecular mechanisms mediating this dual role of FOXQ1 will be discussed in greater detail in Sect. 5.1., below.

## Epithelial-to-mesenchymal transition in tumour metastasis

**Fig. 3:**
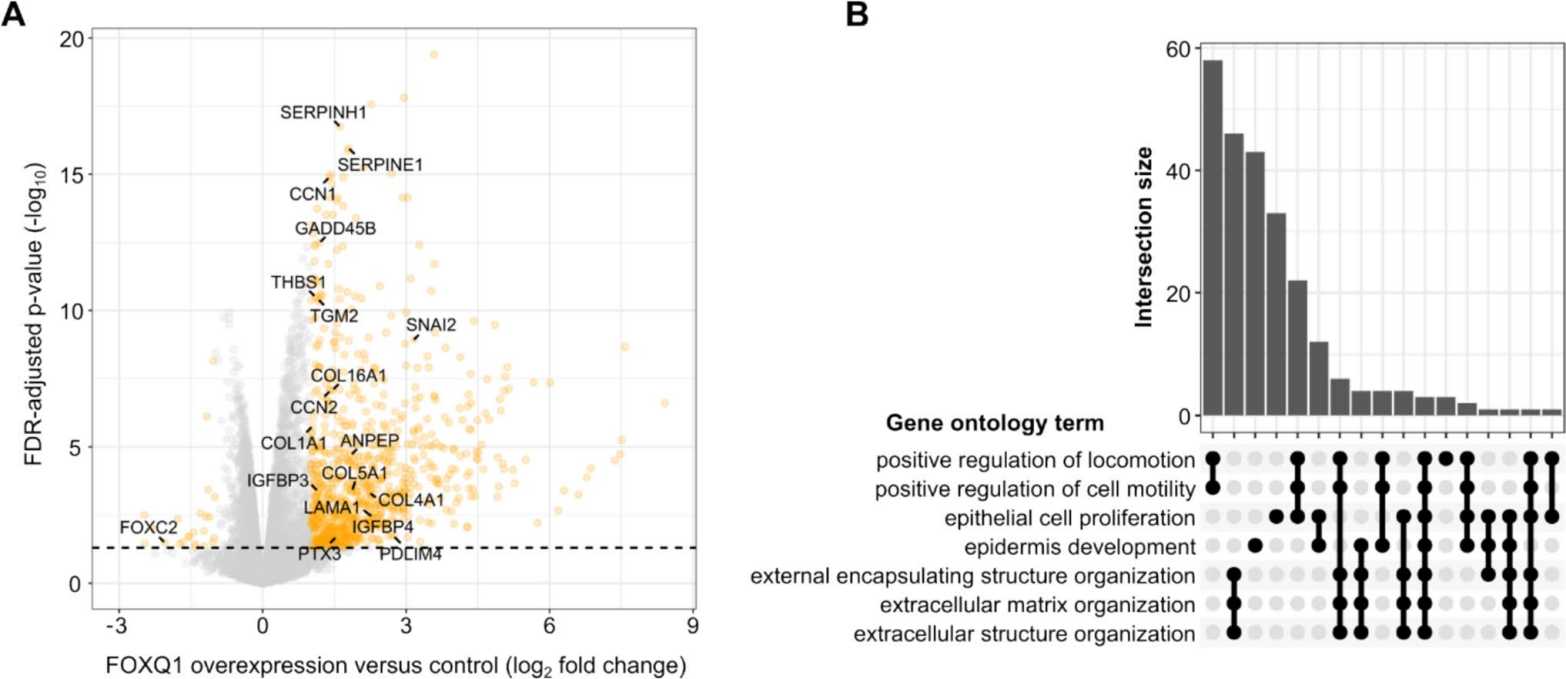
Gene regulation by FOXQ1 in colorectal cancer cells. **A** Volcano plot of differentially expressed genes in HCT116 cells following overexpression of FOXQ1. EMT-associated genes are highlighted. Note, in particular, the significant induction of the core EMT transcription factor SNAI2. FDR: false discovery rate. **B** Corresponding UpSet plot of significantly enriched gene ontology terms (biological process; FDR-adjusted p-values < 0.05) associated with genes induced by FOXQ1 in HCT116 cells. FOXQ1 induces genes involved in cell motility and extracellular matrix remodelling, which are key features of EMT. Data were taken from dataset E-MTAB-12062 [12] and plotted in R 4.4.0

In light of the aforementioned observations, a key question to be resolved is how FOXQ1 affects cancer progression. Although it is likely that FOXQ1 has several functions of which some may be tissue-specific, a common theme that has emerged over the years is that FOXQ1 is a major regulator of epithelial-to-mesenchymal transition (EMT) (Fig. 3). EMT is a process by which epithelial cells temporarily acquire a mesenchymal-like phenotype, which enables them to leave the epithelial layer and promote tissue morphogenesis during embryonic development as well as wound repair after injury [38, 39]. In the context of cancer biology, the same mechanism allows cancer cells to migrate out of the primary tumour, enter the circulation, and form remote metastases in other organs. Moreover, the transformation of tumour cells by EMT is frequently accompanied by the resistance to therapeutic drugs, the acquisition of stem cell features, and possibly tissue fibrosis [40, 41]. Because of this, EMT is associated with disease progression in most cancers, and represents a possible point of intervention for cancer treatment [42].

The EMT of cancer cells is typically initiated by growth factors, cytokines, and chemokines abundant in the tumour microenvironment [43]. Prominent examples of such EMT-inducing factors include the inflammatory cytokines interleukin (IL)−1β and tumour necrosis factor (TNF)-α, and especially the transforming growth factor (TGF)-β1. Downstream of these soluble factors, EMT is coordinated by tissue and context-specific signalling networks whose complexity remains incompletely understood [44]. Nonetheless, some core concepts have emerged over the years. One of these is that cells undergoing EMT pass through multiple intermediary states controlled by specific sets of transcription factors [45]. Although many of these transcription factors differ between individual conditions and cell types [44], there are several proteins that are considered common EMT transcription factors, especially the zinc finger transcription factors SNAI1 and SNAI2 (also referred to as Snail and Slug), ZEB1 and ZEB2, and the basic helix-loop-helix transcription factor TWIST1 [46]. Collectively, these proteins drive a transcriptional program that alters cell adhesion, polarity, and motility towards a mesenchymal phenotype.

Additionally, various other transcription factors are known to contribute to EMT, although their action may be more context-specific [44, 45]. In particular, members of the SOX transcription factor family [47], AP-1 [48], and some NF-κB-associated transcription factors [49] have been implicated in promoting EMT in different types of cancers. Several FOX family transcription factors are involved in EMT as well. For example, FOXOs, FOXCs, and FOXAs, as well as the prominent oncogene FOXM1, have been identified as regulators of tumour cell EMT [50–55]. Considering the high level of conservation and functional redundancy within the FOX family, it is therefore not surprising that FOXQ1 can contribute to EMT as well.

### The contribution of FOXQ1 to the epithelial-to-mesenchymal transition of cancer cells

Shortly after its initial discovery as a possible oncogene, several groups independently reported that FOXQ1 promotes EMT in various types of cancer cells [26, 33, 56, 57]. These include major types of carcinomas, including gastric [58, 59], colorectal [23, 24, 33], lung [26, 60, 61], and breast cancer [9, 56, 62]. Across multiple studies, the induction of FOXQ1 in cell lines derived from different types of cancer caused morphological changes as well as increased cell motility, consistent with EMT, whereas FOXQ1 depletion had the opposite effect. Moreover, FOXQ1 induction promoted other features typically associated with EMT, including the formation of remote metastases in mouse xenograft models, resistance to apoptosis caused by chemotherapeutic drugs, and the increased expression of cancer stem cell markers [22, 33, 56, 63]. On the molecular level, EMT is characterised by a loss of cell adhesion molecules, mainly E-cadherin, and the concomitant gain of mesenchymal markers such as N-cadherin. These changes can be triggered by different morphogens, in particular TGF-β1, and are thought to be an essential prerequisite for tumour metastasis [64, 65]. Interestingly, it has been suggested that FOXQ1 is critically involved in the induction of EMT by TGF-β [24, 56, 57]. Treatment of colorectal and mammary epithelial cell lines with TGF-β induced *FOXQ1* expression, suggesting that FOXQ1 may be a downstream effector of this cytokine in cancer cells. Consistent with this notion, depletion of FOXQ1 inhibited the TGF-β-dependent EMT of mouse mammary epithelial cells [56] and the invasion of colorectal cancer cells into Matrigel substrate [24].

**Fig. 4:**
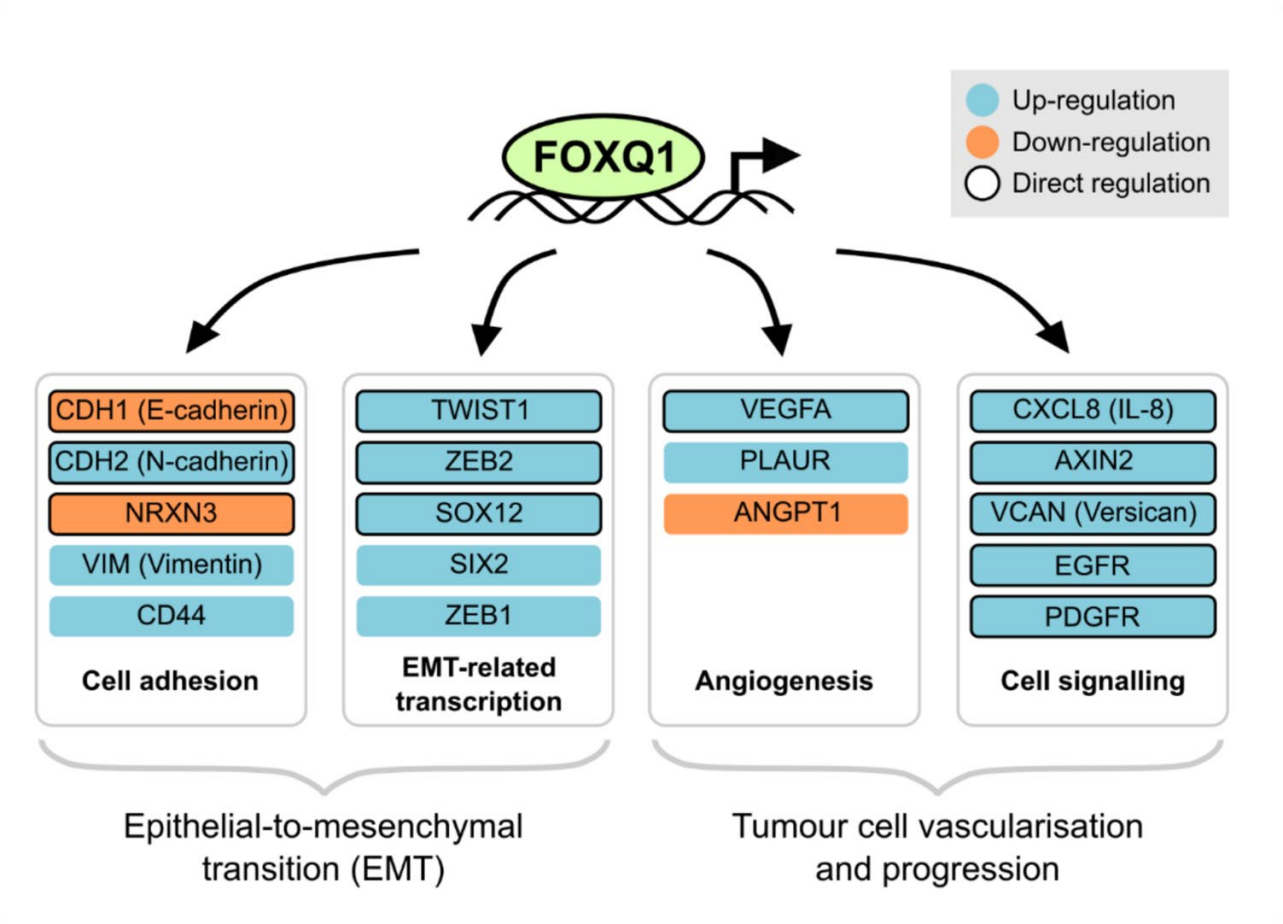
Target genes of FOXQ1. Selected target genes of FOXQ1 that are involved in EMT and tumour progression are shown. Targets with evidence for direct regulation by FOXQ1 (e.g., by ChIP or promoter assays) are highlighted with black outlines

FOXQ1 directly controls the expression of several EMT-associated genes (Fig. 4). It was shown that FOXQ1 engages the E-cadherin and N-cadherin promoters in model cell lines and inversely regulates their transcriptional activity, i.e., it represses E-cadherin and induces N-cadherin [36, 56]. Apart from the direct regulation of cadherin expression, induction of FOXQ1 in cancer cells is associated with an increase of other EMT markers such as vimentin and fibronectin [56, 66, 67]; however, it is likely that these effects are indirect. High-throughput analyses of the FOXQ1-dependent transcriptome have shown that FOXQ1 regulates the expression of potentially hundreds of genes involved in EMT, including other transcription factors [9, 12, 67]. For example, FOXQ1 directly induces the expression of TWIST1 and ZEB2 [23, 68, 69], two well-known EMT-associated transcription factors that also repress E-cadherin. In hepatocellular carcinoma, ZEB2 was shown to be essential for FOXQ1-dependent tumour metastasis [68]. Similarly, the transcription factor SOX12 is a direct transcriptional target of FOXQ1 in HCC cells [70]. SOX12, in turn, controls the expression of TWIST1 and FGFBP1, which were found to be critical for tumour metastasis in this type of cancer.

As can be seen from the preceding examples, EMT is governed by highly complex, context-dependent signalling networks, and determining the contribution of individual transcription factors to this process remains a major technical challenge [44, 71]. Nonetheless, a pooled loss-of-function experiment of putative EMT regulators in breast cancer cell lines supports a critical role of FOXQ1 in EMT [45]. Upon loss of *FOXQ1*, cells undergoing spontaneous EMT were unable to complete their transition to a mesenchymal phenotype. This suggests that FOXQ1 is a bona fide checkpoint regulator of the EMT continuum, at least in some cell types.

### The role of FOXQ1 in the acquired treatment resistance of cancer cells

The EMT of tumour cells frequently results in their increased resistance to radio- and chemotherapy. Mechanisms that have been proposed to explain this phenomenon include the transcriptional inactivation of apoptotic signalling cascades, the induction of transporter proteins that remove drugs from the cells, and the dampening of immune cell activity in the tumour microenvironment [40]. Many of these effects are mediated by EMT transcription factors such as SNAI1 and ZEB1 [72–74], suggesting that FOXQ1-dependent gene transcription may similarly contribute to treatment resistance. Indeed, Mitchell and colleagues observed a considerable overlap between genes regulated by FOXQ1 and SNAI1 in mammary epithelial cells [75]. However, while these targets included numerous genes involved in EMT, the authors did not identify any specific genes that would explain the chemoresistance conferred by FOXQ1.

In the context of CRC, FOXQ1-dependent radioresistance has been linked to the protein deacetylase SIRT1 [76], which was previously found to be a direct transcriptional target of FOXQ1 [77]. Yang et al. reported that radioresistant CRC cell lines derived by extended irradiation exhibited increased *FOXQ1* levels, and that depletion of FOXQ1 reversed their radiation resistance [76]. These authors attributed the effect of FOXQ1 to the regulation of SIRT1 expression, which may promote cancer cell stemness and radioresistance via the activation of the transcription co-factor β-catenin. Similarly, it has been reported that FOXQ1 confers radioresistance in TNBC cell lines [78]. In these cells, FOXQ1 cooperated with the adapter protein RAPH1 in activating STAT3 signalling, which was found to be essential for the resistance to irradiation (see also Sect. 5.3., below).

Finally, the FOXQ1-induced chemoresistance of mammary epithelial cells may be mediated by signalling via platelet-derived growth factors (PDGF). Meng and colleagues observed that FOXQ1 directly induces the receptor tyrosine kinases PDGFRα and PDGFRβ [69]. Knock-down of PDGFRs or inhibition of PDGF signalling sensitised mammary epithelial cells overexpressing FOXQ1 to the treatment with the chemotherapeutic drugs doxorubicin and paclitaxel, and reduced tumour growth in xenograft models *in vivo*. Taken together, these observations suggest that FOXQ1 contributes to the treatment resistance of cancer cells not only indirectly via the induction of EMT, but also through the direct control of mechanisms that make tumour cells resistant to different types of treatments.

### Promotion of tumour vascularisation by FOXQ1

Aside from altering cancer cell properties, tumour metastasis also requires the formation of new blood vessels that act as passageways to distant organs. Neovascularisation can occur either through the recruitment of endothelial cells or vascular mimicry of the cancer cells themselves, and both of these processes are controlled by FOXQ1 as well. FOXQ1 regulates the expression of angiogenic factors such as VEGF, CCL2, and ANGPT1 in cancer cells [22, 79], and consistently, conditioned media from FOXQ1-expressing cells are sufficient to enhance angiogenesis and blood vessel formation by endothelial cells *in vitro* [24, 79]. These effects appear to be at least in part mediated by the FOXQ1 target gene TWIST1. In colorectal cancer cells, depletion of TWIST1 inhibited the CCL2-dependent recruitment of macrophages [79], which is known to be essential for efficient neovascularisation [80]. On the other hand, Luo and colleagues observed that FOXQ1 enhances the vascular mimicry of nasopharyngeal cancer cells [81]. Vascular mimicry in these cells required the activation of the EGF receptor, which these authors identified as a direct transcriptional target of FOXQ1. Consequently, EGFR inhibition counteracted FOXQ1-dependent tumour growth, vascularisation, and metastasis in mouse xenograft assays, and this beneficial effect was further enhanced by co-treatment with the VEGF receptor inhibitor sunitinib [81]. Collectively, FOXQ1 appears to regulate converging angiogenic transcriptional programs that allow cancer cells to connect to the circulation and thereby disseminate to remote locations in the body.

## Specific regulation of gene expression by FOXQ1

**Fig. 5:**
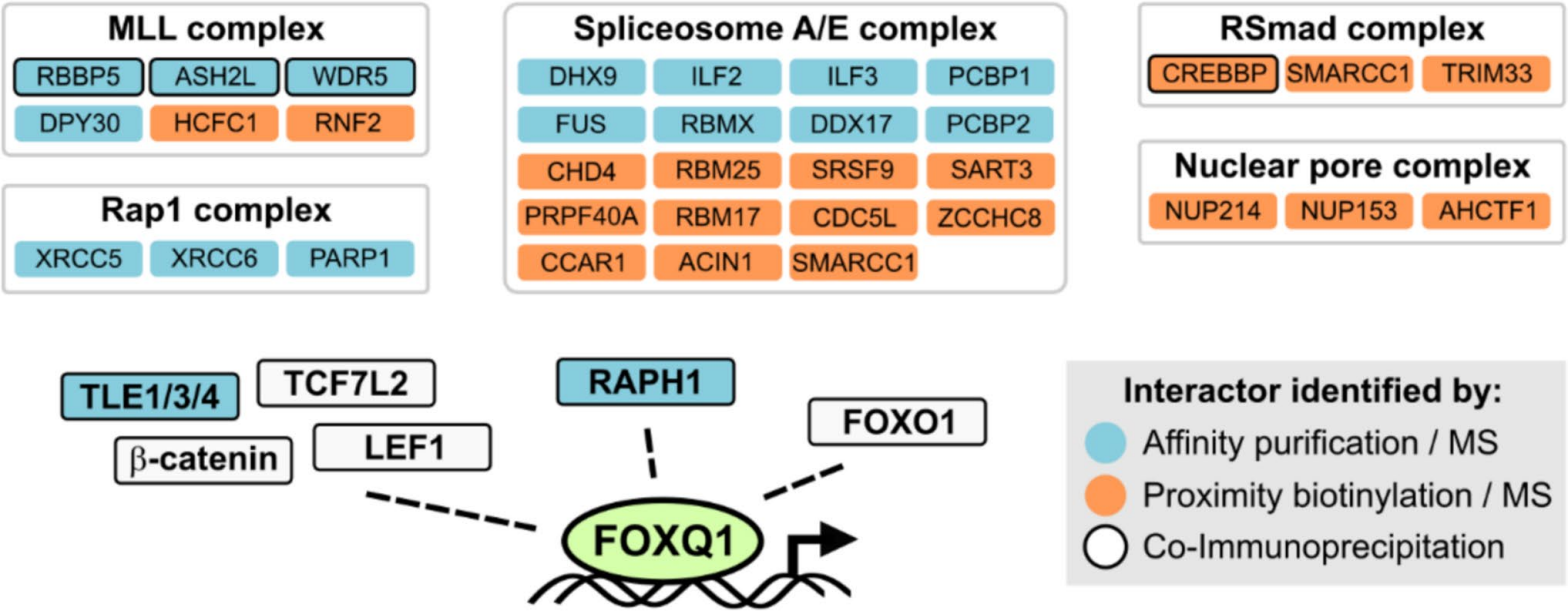
Interactors of FOXQ1. Components of large protein complexes are grouped in grey boxes. MS: mass spectrometry

Chromatin immune-precipation (ChIP)-sequencing as well as RNA-sequencing assays indicate that FOXQ1 can bind to thousands of sites in the human genome and regulate the transcription of as many genes [9, 12, 67]. Analysis of these transcriptional programs supports that FOXQ1 broadly controls cell migration and differentiation, consistent with its role in the EMT of cancer cells (Fig. 3). However, the DNA recognition motif of FOXQ1 is highly similar to most of the other FOX family members, including known tumour suppressors such as FOXF and FOXO proteins [82] (Fig. 1D). This raises the question how specific gene regulation by FOXQ1 can be achieved. Like all transcription factors, FOXQ1 requires various co-factors to fulfil its functions. Large-scale proteomics studies have identified hundreds of putative FOXQ1 interactors in model cell lines [9, 12, 83], which include components of large protein complexes such as the MLL DNA methylation complex, the spliceosome, and the RSmad complex that regulates TGF-β-dependent gene transcription (Fig. 5). Most of these potential co-factors seem to be shared by other FOX family members [83, 84], and functionally important interactors may elude detection in high-throughput assays, especially since all of the assays published to date were done in non-cancer cell lines. Despite these limitations, several studies have discovered FOXQ1 interactors that are relevant for its activity in cancer, which will be discussed below.

### Reciprocal regulation of FOXQ1 and β-catenin/TCF signalling

Based on the curious finding that FOXQ1 acts as a tumour suppressor in melanomas, Bagati et al. hypothesised that cell type-specific interactors determine its function in tumour cells, and identified β-catenin as a FOXQ1-associated protein [36]. β-catenin is best known as an activating co-factor of TCF/LEF family transcription factors in the Wnt signalling pathway. Upon activation of the pathway, β-catenin binds to TCF/LEF, which leads to the inactivation of TLE family repressors that are associated with these transcription factors and thereby to the induction of target gene transcription [85]. TLE proteins have been previously shown to suppress the activity of FOXG1 [86] and were identified as possible FOXQ1 interactors in high-throughput proteomics assays [83, 84], suggesting that a similar transcriptional switch mechanism may also exist in FOX transcription factors. Indeed, overexpression of β-catenin in melanoma cells displaced TLE from FOXQ1 at the N-cadherin promoter and thereby activated N-cadherin expression [36]. This was largely sufficient to override the tumour suppressor function of FOXQ1 in melanoma cells, indicating that the differential regulation of just one gene may decide the context-dependent role of FOXQ1 in cancer. It remains to be seen if the proposed mechanism also applies to other types of cancer in which FOXQ1 may act as a tumour suppressor, such as HER2-positive breast cancer [35], or whether it is specific to melanomas. In fact, in a follow-up study, Bagati and colleagues reported that FOXQ1 directly regulates the expression of the transcription factor MITF, which is a key regulator of melanocyte differentiation [87]. MITF itself acts as a tumour suppressor in melanomas by inhibiting EMT and cell invasion [88, 89], suggesting that the effect of FOXQ1 on melanoma progression is controlled by multiple converging mechanism that may not occur in other cell types.

Interestingly, FOXQ1 interacts not only with TCF/LEF-associated co-factors, but also with TCF/LEF themselves. Our group had previously observed that FOXQ1 strongly activates the oncogenic Wnt/β-catenin signalling pathway [90], which prompted us to investigate the underlying mechanisms. We found that FOXQ1 binds to TCF7L2 and LEF1 (two of the four TCF/LEF family transcription factors) independently of β-catenin, and stabilises the interaction between β-catenin and TCF/LEF [12]. This interaction occurred via the C-terminus of FOXQ1, but surprisingly, deletion of the C-terminus did not reduce the activity of FOXQ1 in Wnt pathway reporter assays. Instead, we observed that FOXQ1 differentially regulates the expression of major Wnt pathway target genes, and synergises with Wnt signalling in imposing an EMT-associated transcriptional program on colorectal cancer cells. Activating mutations in the Wnt pathway are the initiating event in most cases of CRC, and mounting evidence implicates Wnt signalling as a major contributor to EMT in various cancers [91]. This suggests that the induction of FOXQ1 in CRC may shift the Wnt transcriptional response from stem cell maintenance and proliferation to aberrant cell differentiation and migration.

### Regulation of FOXQ1 activity by KMT2/MLL

Another critical interactor of FOXQ1 is RBBP5, a core component of the KMT2/MLL methyltransferase core complex [9]. Mitchell and colleagues observed that FOXQ1 binds to RBBP5 in different types of cancer cells, and that it recruits the KMT2A/MLL1 catalytic subunit to the promoters of EMT-associated genes, thereby inducing their transcription. Accordingly, depletion of RBBP5 or disrupting the RBBP5-binding interface in the forkhead box of FOXQ1 was sufficient to reduce the induction of FOXQ1-driven EMT in mammary epithelial cells. Thus, the KMT2/MLL complex appears to be a critical co-factor for the activation of gene expression by FOXQ1. Interestingly, these authors also observed that overexpression of FOXQ1 in mammary epithelial cells reduced the expression of numerous genes, and the repression of gene transcription by FOXQ1 appeared to be largely independent of RBBP5 [9]. Instead, transcription suppression is mediated by the C-terminus of FOXQ1 [12], which may recruit transcriptional repressors such as TLE family proteins [36] and other, as yet unknown interactors.

### Functional interaction of FOXQ1 with RAPH1 and FOXO1

Other proteins that were found to interact with FOXQ1 are RAPH1 and the related transcription factor FOXO1 [18, 78]. RAPH1 is a poorly characterised adapter protein that contributes to lamellipodia formation and cell adhesion. Using mass spectrometry in cancer cells lines, Liu et al. identified RAPH1-v3, a splice variant of RAPH1 that localises to the nucleus and is enriched in radioresistant TNBC cells, as a FOXQ1 interactor [78]. FOXQ1 synergised with RAPH1-v3 in promoting TNBC cell proliferation, migration, and apoptosis resistance. Further investigation revealed that the effect of RAPH1-v3 in cancer cells is mediated at least in part by increasing the activity of the transcription factor STAT3, which is an established oncogene in TNBC. Based on these findings, the authors suggest that FOXQ1 recruits RAPH1-v3 to STAT3 binding sites, thereby boosting the transcriptional activity of STAT3 [78]. Finally, Cui and colleagues observed that FOXQ1 binds to the forkhead box domain of FOXO1 and inhibits the transcription of FOXO1 target genes in hepatocytes [18]. Although these authors explored the function of FOXQ1 in the context of glucose metabolism, the findings may have important implications for cancer biology. FOXO transcription factors are well-known inhibitors of tumour progression, and FOXO1 has been shown to suppress EMT across various types of cancer [92–94]. Thus, FOXQ1 may reinforce the EMT of cancer cells by inactivating tumour suppressor proteins.

Collectively, these observations highlight important interactors of FOXQ1 that may be critical for its function in cancer cells. Going forward, it will be of considerable interest to determine if these interactors are specific for FOXQ1, whether these interactions are dependent on the type of cells, and how these interactions can be targeted for the treatment of cancers.

## Regulation of FOXQ1 mRNA and protein levels in cancer cells

**Fig. 6:**
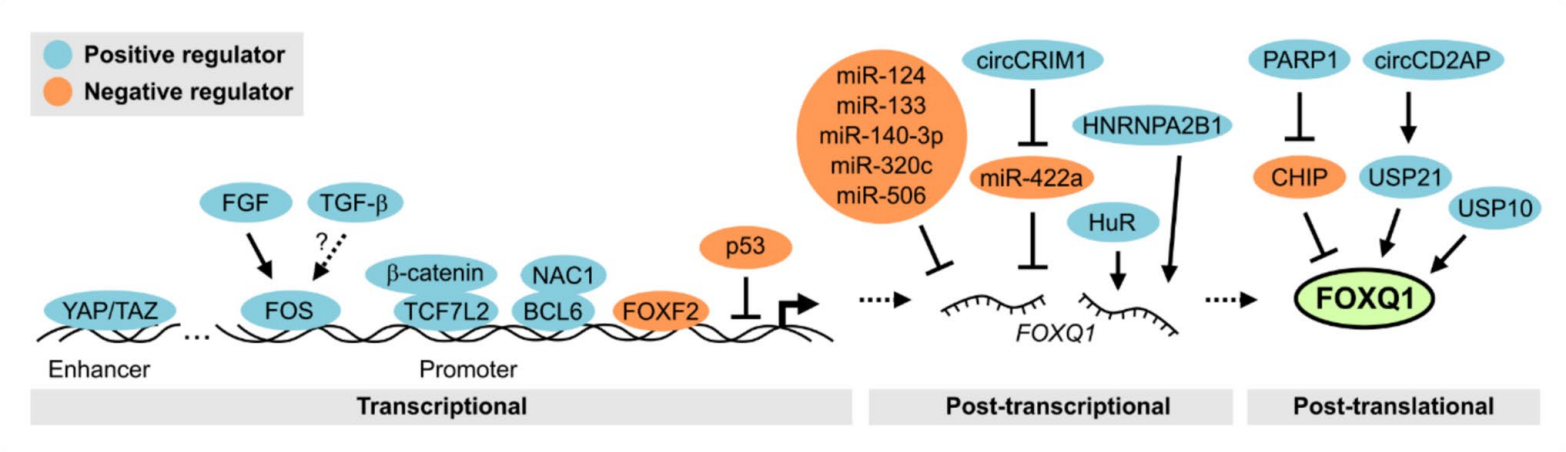
Regulators of FOXQ1 expression and stability

Having discussed how FOXQ1 controls gene expression in cancers, another relevant question to address is how the abundance of FOXQ1 itself is controlled in tumour cells. Several studies, discussed in the following sections, have outlined possible mechanisms that may drive the induction and accumulation of FOXQ1 in cancer (Fig. 6).

### Transcriptional regulation of FOXQ1 gene expression

As discussed in the previous section, FOXQ1 collaborates with TCF/LEF family transcription factors to boost the induction of Wnt pathway target genes. Interestingly, FOXQ1 itself is a target of Wnt signalling. Christensen and colleagues identified a TCF7L2 binding site in the FOXQ1 promoter, and showed that activation of the Wnt signalling pathway leads to increased FOXQ1 expression levels in different cell lines [25]. Moreover, FOXQ1 expression in multiple CRC cell lines correlated with the intrinsic Wnt pathway activity of these cells. This suggests that chronic activation of Wnt signalling in CRC leads to sustained high levels of FOXQ1, which in turn synergises with Wnt signalling to drive a metastasis-associated transcriptional program. Of note, FOXQ1 has been used as a marker for intestinal epithelial stem cells [95], which reside in an active Wnt signalling niche, and was found to be one of very few Wnt pathway target genes expressed in mouse foetal intestinal epithelia [96]. Thus, low FOXQ1 levels maintained by physiological Wnt pathway activity may contribute to the homeostasis of epithelial stem cells in the gut, whereas aberrant FOXQ1 induction results in uncontrolled cell differentiation. Interestingly, it appears that FOXQ1 is similarly involved in epithelial stem cell maintenance in the prostate. Kirk and colleagues recently reported that Foxq1 may contribute to prostate epithelial regeneration in mice, a process that is marked by strong activation of Wnt signalling [97]. Although this study did not explore the connection between FOXQ1 and Wnt signalling in detail, it seems that the Wnt-dependent regulation of FOXQ1 levels has important implications beyond the biology of cancer.

FOXQ1 levels can also be increased through other cancer-associated signalling pathways, such as FGF and TGF-β signalling [24, 98]. Lin et al. reported that activation of the FGF receptor increased the expression FOXQ1 in breast cancer cell, which was mediated by the common signal transducers MEK/ERK [98]. The authors further showed that following activation of these transducers, FOS/AP-1 binds to the FOXQ1 promoter and induces its transcription. In line with these observations, TGF-β was found to increase the expression of FOXQ1 in CRC cells [24]. Although these authors did not explore any downstream signalling events, it has been shown that the activation of SMAD family transcription factors by TGF-β synergises with FOS/AP-1 in promoting the invasiveness of breast cancer cells [99]. This raises the intriguing possibility that FGF and TGF-β cooperate in inducing FOXQ1 expression and thereby promote tumour progression.

Other transcription factors known to regulate FOXQ1 expression include YAP/TAZ and BCL6 [100, 101]. The related proteins YAP and TAZ contribute to the transcriptional dysregulation of many types of cancer. In a screen of dysregulated genomic regulators in patient-derived CRC organoids, Della Chiara and colleagues identified a distal enhancer of FOXQ1 that was bound by YAP/TAZ, and showed that YAP and FOXQ1 were co-expressed in tissues from patients with CRC [100]. Moreover, Gao et al. reported that a BCL6 induces the expression of FOXQ1 in ovarian cancer cells, which is mediated by the transcription co-factor NAC1 [101, 102]. These authors identified multiple BCL6 binding sites in the FOXQ1 promoter, of which one was required for FOXQ1 induction.

To gain further insight into the regulation of FOXQ1 expression, our group recently performed a proximity proteomics-based screen of proteins associated with the human FOXQ1 promoter [103]. Focusing on a highly active region of the promoter close to the transcriptional start site, we found that the prominent tumour suppressor p53 associates with this region and negatively regulates the expression of FOXQ1. p53 depletion in various cancer cell lines modestly de-repressed FOXQ1, which was sufficient to enhance the expression of some cancer-related FOXQ1 target genes. Additionally, we observed that loss of p53 synergises with the activation of Wnt signalling in driving the induction of FOXQ1 in CRC cells [103]. Loss-of-function of p53 occurs in more than half of all cases of cancer [104], and de-repression of FOXQ1 appears to be one of its many pathophysiology consequences. Another transcriptional repressor of FOXQ1 is the related protein FOXF2, and in fact, FOXQ1 and FOXF2 were found to trans-repress each other in breast cancer cells as well as during embryonic development [62, 105]. Kang et al. showed that FOXF2 recruits an NCOR1/HDAC3 complex to the FOXQ1 promoter to de-acetylate nearby histones and thereby silence its transcription [62]. In contrast, the repression of FOXF2 by FOXQ1 was reportedly not mediated by the same mechanism. Considering that the DNA recognition motifs of FOXQ1 and FOXF2 are virtually identical [82], this study provides an interesting example of the critical role of transcription co-factors in shaping the function of transcription factors as oncogenes or tumour suppressors.

### Regulation of FOXQ1 mRNA and protein stability

Besides transcriptional regulation, the levels of FOXQ1 in cancer cells are also determined by post-transcriptional and post-translational mechanisms (Fig. 6). FOXQ1 is a target of microRNAs (miRNAs), a class of small non-coding RNAs whose main function is the silencing of complementary mRNAs. Multiple studies reported silencing of FOXQ1 by various miRNAs in different types of cancer, and collectively suggest that post-transcriptional inhibition of FOXQ1 may prevent EMT and tumour progression [60, 106–109]. Of particular interest, Hong et al. outlined a detailed post-transcriptional mechanism of FOXQ1 induction in nasopharyngeal carcinoma (NPC) cells [110]. The authors observed that another class of non-coding RNA, namely the circular RNA circCRIM1, is over-expressed in highly metastatic NPC cells. circCRIM1 was found to act as a sponge for miR-422a, an inhibitor of FOXQ1. Accordingly, circCRIM1 increased FOXQ1 levels and thereby promoted NPC cell EMT and metastasis in xenograft models. Conversely, FOXQ1 mRNA appears to be stabilised by the nuclear ribonucleoprotein (hnRNP) HNRNPA2B1 [111]. HNRNPA2B1 was found to bind to FOXQ1 mRNA in squamous cell carcinoma cells, presumably via a methylated consensus RNA motif, and increased FOXQ1 transcript and protein levels.

Additionally, recent studies have uncovered mechanisms that control FOXQ1 protein stability. Wu and colleagues identified FOXQ1 as a substrate of the E3 ubiquitin ligase CHIP, which facilitates its proteasomal degradation [112]. PARP1 disrupted the interaction of FOXQ1 and CHIP in ovarian cancer cells, which increased the protein levels of FOXQ1. PARP proteins have multiple important roles in cancer, including the regulation of DNA repair, and PARP inhibitors are in clinical use for the treatment of several types of cancer [113]. Interestingly, the PARP inhibitor niraparib attenuated tumour growth in a mouse xenograft model with ovarian cancer cells expressing FOXQ1 [112], suggesting that PARP inhibition may have the additional benefit of counteracting FOXQ1 in cancer. The proteasomal degradation of proteins can also be prevented by ubiquitin-specific peptidases (USPs). Wang and colleagues reported that USP21 stabilises FOXQ1 in bladder cancer cells [114]. USP21, in turn, was stabilised by circCD2AP, a circular RNA that was found to be increased in bladder cancer. Accordingly, circCD2AP promoted the EMT and migration of model cell lines, which was mediated by FOXQ1. Zhao et al. observed that FOXQ1 is similarly de-ubiquitinated by USP10 in human kidney cells, which leads to the stabilisation of the protein [115]. Although these authors investigated the role of USP10-dependent regulation of FOXQ1 in the context of acute kidney injury, these findings may have implications for cancer biology as well. USP10 is involved in the regulation of numerous cancer-related proteins such as c-MYC and p53, and has been posited as an oncogene or tumour suppressor in different types of cancer [116]. In light of these findings, it appears that part of its function in cancer is the stabilisation of FOXQ1, which would be interesting to explore further. Taken together, these studies indicate that the expression and stability of FOXQ1 are tightly controlled on multiple levels, and suggest possible explanations for the tissue-specific induction of FOXQ1 in cancer.

### Regulation of FOXQ1 activity by post-translational modification

Apart from the mechanisms outlined above, there is also some evidence that the activity of FOXQ1 can be regulated by post-translational modifications (PTM). Liu and colleagues recently reported that treatment of HCC cells with the multi-kinase inhibitor sorafenib resulted in the phosphorylation of FOXQ1 at the serine residue 284 [117]. The authors identified JNK1 as a FOXQ1 kinase, and observed that phosphorylation of FOXQ1 promoted the induction of ETHE1, a persulfide dioxygenase involved in the regulation of ferroptosis. Accordingly, FOXQ1 inhibited the sorafenib-induced ferroptosis of HCC cells, which was in part mediated by ETHE1. Phosphorylation of FOXQ1 at S248 has also been observed in high-throughput proteomics assays of breast cancer samples [118], suggesting that this PTM may be relevant for other types of cancer as well. It will therefore be of interest to determine the mechanisms by which phosphorylation of FOXQ1, or other types of PTM such as acetylation, affect its activity.

### Regulation of FOXQ1 levels by the tumour microenvironment

While many of the factors that affect the abundance and activity of FOXQ1 can be explained by genetic or epigenetic changes in cancer cells, there is also some evidence that the tumour microenvironment contributes to the regulation of FOXQ1 levels. Luo and colleagues reported that patient-derived cancer-associated fibroblasts (CAF) increased the abundance of FOXQ1 in HCC cell lines [119]. Interestingly, these authors observed a feed-back loop mediated by the FOXQ1 target gene NDRG1 that induced various chemokines including CCL26, which promoted the migration of additional CAFs to the site of the tumour. Moreover, immune cells may control FOXQ1 levels in cancer cells as well. It has been shown that *FOXQ1* expression is increased in gastric cancer cells co-cultured with the monocytic cell line THP-1, and that high numbers of monocytes are associated with elevated FOXQ1 levels in samples from patients with gastric cancer [59]. Notably, FOXQ1 may also be induced in macrophages and increase their directed migration, as was shown in the context of skin inflammation [120]. Thus, it is conceivable that FOXQ1 has additional roles in non-epithelial cells that may be relevant for cancer biology.

Taken together, these studies suggest that FOXQ1 levels in cancer may at least partially be controlled by the tumour-associated stroma, and that FOXQ1-dependent gene regulation in turn shapes the tumour microenvironment, which is supported by *in silico* analyses of public cancer data [121].

## FOXQ1 as a potential therapeutic target in cancer

As discussed in the preceding sections, FOXQ1 is involved in EMT and metastasis in several types of cancer, and there is mounting evidence from preclinical models that interfering with FOXQ1-mediated functions in cancer cells can slow down or even reverse tumour progression. It is therefore worthwhile to consider if FOXQ1 could be targeted for the treatment of cancers, especially since evidence from knock-out mice suggests that FOXQ1 is largely dispensable for normal tissue homeostasis in the adult organism [14, 15]. Transcription factors have long been considered undruggable because their often highly disordered structure does not offer obvious binding pockets for small molecule inhibitors. However, recent advances in chemical biology and high-throughput drug discovery assays have led to substantial progress in the field, and various compounds targeting other transcription factors such as p53 and the oestrogen receptor have now entered clinical trials [122]. Moreover, newly discovered small molecules inhibitor of FOXO1 and FOXM1 have shown efficacy in preclinical cancer models [123–125], demonstrating the feasibility of targeting FOX transcription factors *in vivo* as well. Thus, it is realistic that assays such as small molecule inhibitor screens could uncover compounds that block the interaction of FOXQ1 with critical transcription co-factors such as, for example, RBBP5, and thereby modulate the expression of FOXQ1 target genes.

Interestingly, while no such screen has been reported to date, a similar experimental approach identified an inhibitor of the RNA binding protein HuR that decreased the abundance of FOXQ1 mRNA in breast cancer cells [126]. HuR levels are increased in several types of cancer, and it is thought to promote tumour progression by stabilising the transcripts of various oncogenes [127]. Wu et al. found that the FOXQ1 transcript is one of the mRNAs bound and stabilised by HuR, and that the effect of HuR on breast cancer cell migration was at least in part mediated by FOXQ1 [126]. The small molecule inhibitor KH-3 disrupted the binding of HuR to FOXQ1 mRNA, which substantially decreased FOXQ transcript abundance. Thus, targeting FOXQ1 mRNA may be an alternative approach for inhibiting FOXQ1, at least in cancers with high HuR levels. Another method for disrupting protein function is the use of proteolysis targeting chimeras (PROTACs). PROTACs are protein-binding compounds that recruit E3 ubiquitin ligases to specific target proteins, thereby inducing their proteasomal degradation. Several PROTACs have entered clinical trials for cancer treatment, including compounds targeting transcription factors [128]. PROTACs that degrade FOXM1 have already been generated and validated in preclinical models [129], which again establishes the feasibility of exploring their use as inhibitors of FOXQ1.

## Conclusions and future directions

It is increasingly clear that the transcription factor FOXQ1 contributes to tumour progression and dissemination in several types of cancer, especially carcinomas of the gastrointestinal tract. Several recent studies have outlined key mechanisms by which FOXQ1 drives EMT and tumour metastasis and have identified important regulators that control FOXQ1 levels and FOXQ1-dependent gene transcription. These discoveries open up several possible avenues for targeting FOXQ1 in cancer, which could lead to the development of new drugs for the treatment of cancers. An important next step towards this aim will be the development of specific inhibitors that decrease FOXQ1 abundance or block the interaction of FOXQ1 with its co-factors. In light of evidence from preclinical studies, this may be sufficient to render FOXQ1 dysfunctional and thereby prevent tumour progression. An alternative strategy may be to “repurpose” FOXQ1 as a tumour suppressor, considering its tumour-inhibiting role in melanomas. To achieve these goals, several open questions remain to be addressed:

1. What determines the abundance of FOXQ1 in different types of cancer? FOXQ1 expression is silenced in most adult tissues, yet its transcription is frequently induced in cancers. Are there any common (epigenetic) mechanisms that drive its induction, and how does the tumour microenvironment contribute to the expression of FOXQ1?
2. Why are high FOXQ1 levels associated with improved survival in some cancers? Evidence from melanoma suggests that FOXQ1 can suppress the EMT of melanocytes. Is this also the case in other types of cancer such as HER2-positive breast cancer and prostate cancer, and are the mechanisms of tumour suppression shared between these diseases?
3. Can FOXQ1 be safely and effectively targeted for cancer therapy? Although Foxq1-deficient mice are viable and fertile, suggesting that its loss-of-function is tolerable, FOXQ1 may have important functions in specific cell types in the adult organism. Thus, the safety profile of potential FOXQ1-targeting therapies needs to be carefully evaluated before their clinical application.
4. To what extent are the functions of FOXQ1 unique? Many forkhead box transcription factors share a similar DNA-binding motif and recruit comparable sets of transcription co-factors. Are there any specific FOXQ1 target genes, or is its role in cancer redundant with other FOX family members?
5. Do post-translational modifications have a major impact on the activity of FOXQ1? It is well established that other forkhead box family members are controlled by PTMs such as phosphorylation and acetylation, but the post-translational regulation of FOXQ1 has received little attention so far. Do PTMs similarly affect the transcriptional activity and stability of FOXQ1, and could these modifications explain the tissue-specific functions of FOXQ1?

## Data Availability

Gene expression and survival data in fig. 2 are from the The Cancer Genome Atlas Program (TCGA; https://www.cancer.gov/ccg/research/genome-sequencing/tcga). Data were accessed and processed via GEPIA 2 (http://gepia2.cancer-pku.cn/#index) on December 10th, 2024. Gene expression data in fig. 3 are from dataset E-MTAB-12062, available through EBI ArrayExpress (https://www.ebi.ac.uk/biostudies/arrayexpress/studies/E-MTAB-12062).

## Abbreviations and Acronyms

CAF: Cancer-associated fibroblasts
ChIP: Chromatin immuno-precipitation
CRC: Colorectal cancer
EMT: Epithelial-to-mesenchymal transition
FDR: False discovery rate
FOX: Forkhead box
HCC: Hepatocellular carcinoma
miRNA: Micro-RNA
NPC: Nasopharyngeal carcinoma
PDGF: Platelet-derived growth factor
PROTAC: Proteolysis targeting chimera
PTM: Post-translational modification
TCGA: The Cancer Genome Atlas
TGF: Transforming growth factor
TLE: Transducin-like enhancer protein
TNBC: Triple negative breast cancer
TNF: Tumour necrosis factor
USP: Ubiquitin-specific peptidase
